# Editorial: Cyberbullying and Mental Health: An Interdisciplinary Perspective

**DOI:** 10.3389/fpsyg.2021.827106

**Published:** 2022-01-12

**Authors:** Claudio Longobardi, Robert Thornberg, Rosalba Morese

**Affiliations:** ^1^Department of Psychology, University of Turin, Turin, Italy; ^2^Department of Behavioural Sciences and Learning, Linköping University, Linköping, Sweden; ^3^Faculty of Communication, Cultural and Society, Faculty of Biomedical Sciences, Università della Svizzera italiana, Lugano, Switzerland

**Keywords:** cyberbullying, mental health, adjustment (psychology), adolescents, cross cultural

## Introduction

Adolescents are at risk of various forms of peer victimization, particularly in the school context. However, in the last decade, with the development of new technologies and the proliferation of social media among adolescents, the phenomenon of cyberbullying has attracted the attention of researchers, practitioners, and policy makers, considering the impact of cyberbullying victimization on the psychological adjustment and psychophysical integrity of minors.

Knowledge of the phenomenon of cyberbullying is not only a scientific and theoretical curiosity, but also allows appropriate prevention and intervention strategies to be more effective. Although scientific research has identified cyberbullying as a risk factor for adolescent mental health, little is known about the possible mechanisms and mediating factors involved in this relationship. Theoretical models of the relationship between cybervictimization and mental health are underdeveloped, particularly in the emerging field of social neuroscience.

The goal of this Research Topic is to advance current knowledge of the relationship between cybervictimization and mental health, promote an interdisciplinary view of the phenomenon, and identify opportunities for prevention and intervention.

For the Research Topic, 13 contributions with different cultural backgrounds were compiled, including two literature reviews and 11 empirical studies, two of which applied a qualitative approach.

## Literature Review and Theoretical Contributions

In their mini review, McLoughin et al. point out that there is a gap in the literature on how cyberbullying affects brain development. According to the authors, this is an important limitation, as developmental cognitive neuroscience could help us to understand which factors increase the likelihood of an adolescent becoming involved in cyberbullying, as either a victim or an aggressor, and to develop tailored interventions. In particular, the authors emphasize the importance of encouraging longitudinal studies using brain imaging techniques to understand how cyberbullying may affect brain development according to gender and age. The importance of interdisciplinary approaches is also emphasized by Auriemma et al. who propose a theoretical model for understanding the cyberbullying phenomenon based on complex and multifaceted constructs of empathy such as emotional contagion, theory of mind, compassion, prosocial behavior, egocentric bias, and individual traits.

## Empirical Findings: Quantitative Data on Cyberbullying and Developmental Outcomes

Empirical articles have examined the relationship between cyberbullying and mental health in adolescents, pointing to possible mediating mechanisms. Wachs et al. found that high levels of alexithymia tended to mediate the relationship between cyberbullying victimization and measures of self-esteem and Internet addiction in three different countries: Germany, the Netherlands, and the United States.

The paper by Yu et al. from China attempts to expand knowledge of possible mechanisms to explain the relationship between cybervictimization and non-suicidal self-injury. Based on social control theory and the organism-environment interaction model, the authors report that school engagement is a possible mediating factor between cybervictimization and non-suicidal self-injury among adolescents with high sensation seeking.

In a large sample of Chinese adolescents, Chen et al. found that cybervictimization may increase the risk of deviant peer affiliation, which may help to explain the association between cybervictimization and increased drinking behavior among adolescents. In addition, the authors note that the personal growth initiative plays a mediating role. Consistent with the person-environment interaction model, the authors posit that personal growth initiative is a potential protective factor for the indirect effects of cybervictimization on adolescent drinking.

In a large sample of Chinese adolescents, Wang et al. confirm a significant correlation between cybervictimization and Internet addiction, identifying depression as a possible mediating factor. Interestingly, the authors note that positive peer affiliation does not appear to protect adolescents from negative outcomes when they experience high levels of cybervictimization. This suggests the need for further studies on the relationship between cybervictimization and mental health, and on the mediating role of peer relationships, particularly prosocial peer affiliation.

The pandemic situation and lockdowns around the world have created a context in which forms of cybervictimization can proliferate. The paper by Han et al. addresses the relationship between cyberbullying and mental health in the context of the COVID-19 pandemic and specifically targets a rural population of Chinese youth. In the context of the COVID-19 outbreak in 2020, the authors examined the associations between involvement in cyberbullying, resilient coping, and loneliness. They show that resilient coping strategies can reduce the association between cyberbullying and loneliness. Moreover, bullying victims tend to exhibit higher levels of loneliness and lower levels of resilient coping than perpetrators who engage in bullying alone or victims who engage in bullying alone.

The Italian paper by Saladino et al. adds to our knowledge of adolescents' personal cognitions and perceptions of cyberbullying and its consequences. In addition, the authors explain how these data can support cyberbullying prevention and intervention efforts in the school context.

Cyberbullying prevention cannot focus exclusively on victims and aggressors and must consider the entire social scene involved in the dynamics of bullying and cyberbullying. With this in mind, Jungert et al. experimental study addresses potential bystander figures and helps us to better understand when and why youth are motivated to help bullying victims. Research has only recently focused on the bystander figure, but we believe that understanding the factors involved in the predisposition and decision to help a victim of bullying or cyberbullying could have important implications for preventing and counteracting the phenomenon.

Research on the relationship between psychological well-being and cyberbullying has focused predominantly on adolescents, with little evidence on younger students. With this in mind, the brief report by Sidera et al. seeks to expand our knowledge on the relationship between cyberbullying victimization and psychological adjustment in elementary school. The authors report that 14% of the students surveyed had been victims of cyberbullying at least once in the past 2 months, and many of them reported having been victims of traditional bullying as well. The data show that males are at greater risk of being victims of cyberbullying than females, and that the impact of cyberbullying is greater on children who have not also experienced traditional bullying. It is possible that cyberbullying in childhood has different risk factors added to social exclusion (Morese and Longobardi, 2020) and impacts on developmental processes than in adolescence, and future research in this area should be encouraged.

Another stage of the life cycle that appears to be under-researched is adulthood. There is limited research on the relationship between cyberbullying and psychological well-being in adults. In relation to this, Schodt et al. conducted two studies on the relationship between psychological symptoms and involvement in cyberbullying among American adults. In doing so, they attempted to fill a gap in the literature by finding an association between mental health measures and increased risk of involvement in cyberbullying as a victim or aggressor, particularly among men who use social media more. These data appear to differ in part from the literature for adolescents. Therefore, further research on the relationship between mental health and cyberbullying at any developmental stage should be encouraged.

## Empirical Findings: Qualitative Research on Adolescents' Perceptions and Experiences of Cyberbullying

Two interesting qualitative research articles are found within this Research Topic. Li and Hesketh carried out semi-structured interviews with 41 students (12–16 years old) involved in traditional bullying and cyberbullying. The authors found that traditional bullying is more common than cyberbullying, although there is a great deal of overlap between the two types. They developed a conceptual framework which identified a number of risk factors at the organizational and individual levels, pointing to a lack of support from parents and teachers, even when needed, leading to poorer developmental and academic outcomes.

Mishna et al. have also sought to expand current knowledge about how adults, parents, and teachers perceive traditional bullying and cyberbullying. According to the authors, it is important to examine how adolescents and adults (who represent three critical relationship systems in the ecological context of bullying) conceptualize the nature and impact of peer victimization in online and offline contexts in order to identify more accurate and effective prevention and intervention strategies.

## Conclusions

In conclusion, the Research Topic highlights the importance of considering cyberbullying as a risk factor for the psychological adjustment of individuals and adolescents in particular. It is important to increase our knowledge on the relationship between cyberbullying and mental health to understand which areas of individual functioning are affected and which mediating factors are involved. This knowledge will allow us to identify at-risk situations more accurately and implement prevention and intervention strategies more effectively.

The collected contributions point to the need to address and prevent forms of peer victimization, including cyberbullying. Prevention efforts must target all actors involved in the dynamics of bullying and cyberbullying—not only the victims and perpetrators of bullying, but also the observers and the adults (teachers and parents) among their peers. In this respect, the collected research contributions emphasize the importance of making individuals aware of the definition of the phenomenon of cyberbullying and its consequences, starting from the knowledge and personal perceptions that individuals—both adults and minors—develop regarding the phenomenon.

In addition, we believe it is important to increase the scientific knowledge on the relationship between cybervictimization and mental health at different developmental stages, including childhood and adulthood. In connection with this, we emphasize the importance of an interdisciplinary approach when studying the relationship between cyberbullying and psychological adjustment, and we believe that social neuroscience can help expand our knowledge and develop theoretical models that can contribute to prevention and intervention.

## Author Contributions

All authors listed have made a substantial, direct, and intellectual contribution to the work and have approved it for publication.

## Conflict of Interest

The authors declare that the research was conducted in the absence of any commercial or financial relationships that could be construed as a potential conflict of interest.

## Publisher's Note

All claims expressed in this article are solely those of the authors and do not necessarily represent those of their affiliated organizations, or those of the publisher, the editors and the reviewers. Any product that may be evaluated in this article, or claim that may be made by its manufacturer, is not guaranteed or endorsed by the publisher.
